# Quantitative Evaluation of Bacteria Adherent and in Biofilm on Single-Wall Carbon Nanotube-Coated Surfaces

**DOI:** 10.1155/2011/291513

**Published:** 2011-10-05

**Authors:** Fabrizio Pantanella, Francesca Berlutti, Daniele Passeri, Daniela Sordi, Alessandra Frioni, Tiziana Natalizi, Maria Letizia Terranova, Marco Rossi, Piera Valenti

**Affiliations:** ^1^Dipartimento di Sanità Pubblica e Malattie Infettive, Sapienza-Università di Roma, Piazzale A. Moro 5, 00185 Roma, Italy; ^2^Centro di Ricerca per le Nanotecnologie Applicate all'Ingegneria, CNIS, Sapienza-Università di Roma, Piazzale A. Moro 5, 00185 Roma, Italy; ^3^Dipartimento di Scienze di Base e Applicate per l'Ingegneria, Sapienza-Università di Roma, Via A. Scarpa 16, 00161 Roma, Italy; ^4^Dipartimento di Scienze e Tecnologie Chimiche, MINASlab, INFN, Università di Roma Tor Vergata, Via della Ricerca Scientifica, 00133 Roma, Italy

## Abstract

Biofilm is a common bacterial lifestyle, and it plays a crucial role in human health, causing biofilm-mediated infections. Recently, to counteract biofilm development, new nano-structured biomaterials have been proposed. However, data about the antibacterial properties of nano-structured surfaces are fragmentary and controversial, and, in particular, the susceptibility of nano-structured materials to colonization and biofilm formation by bacterial pathogens has not been yet thoroughly considered. Here, the ability of the pathogenic *Streptococcus mutans* and *Pseudomonas aeruginosa* to adhere and form biofilm on surfaces coated with single-wall carbon nanotubes (SWCNTs) was analyzed. Our results showed that the surfaces of SWCNTs-coated glass beads (SWCNTs-GBs) were colonized at the same extent of uncoated GBs both by *S. mutans* and *P. aeruginosa*. In conclusion, our results demonstrate that single wall SWCNTs-coated surfaces are not suitable to counteract bacterial adhesion and biofilm development.

## 1. Introduction

Infectious disease is one of the most important causes of mortality. Despite the great life expectancy related to advanced health care, the increasing numbers of complicated healthcare infections remain a significant public health challenge. A growing body of evidences shows that bacterial biofilm lifestyle is comparatively more common than planktonic one, playing a crucial role in human health despite the therapeutic use of antibiotics [1–3]. Moreover, the problem of biofilm-mediated infections becomes much more severe when biofilm colonizes medical devices and biomaterials [4–6]. 

Therefore, the possibility to counteract bacterial colonization of medical device and biomaterial surfaces represents a crucial issue in human health. In the past few years nanotechnology has broken into medicine like a tsunami involving in these new field researchers with different skills. Nano-structured materials have been recently proposed as a pragmatic approach in the discovery of new biomaterials to counteract bacterial colonization and biofilm development. In particular, the antibacterial activity of carbon nanotubes (CNTs) has been explored. The literature data show that dispersed as well as incorporated CNTs into different polymers exerted antibacterial activity. In particular, CNTs showed bactericidal activity against both Gram-positive and Gram-negative bacteria [7–10] while the antibiofilm activity of CNTs has been demonstrated only for the Gram-negative *Escherichia coli* K12 strain [11]. However, the susceptibility of nano-structured surfaces to colonization and biofilm formation by pathogenic bacteria has not been yet thoroughly considered as well as the efficiency and the effect of the sterilization process on nano-structured surfaces. The underestimation of the potential risk of contamination by bacteria able to adhere and form biofilm on nano-structured surfaces can lead to the unwanted onset of bacterial infections like what happened in the early biomaterial era. 

Here, the ability of two bacterial pathogenic species, that is, *Streptococcus mutans* and *Pseudomonas aeruginosa*, to adhere and form biofilm on surfaces coated with single wall CNTs (SWCNTs) was evaluated. *S. mutans* and *P. aeruginosa* have been chosen as bacterial models for their well-known ability to adhere and grow in biofilm lifestyle and for their implication in human diseases, as discussed elsewhere [12–17]. 

To investigate the ability of bacterial pathogens to colonize SWCNTs-coated surfaces, conventional methods such as crystal violet (CV) staining method, which stains both bacterial cells and matrix, BacLight LIVE/DEAD able to detect living and death cells into biofilm as well as atomic force microscopy (AFM) were employed [11, 18, 19]. However, a fundamental prerequisite in studying bacterial adhesion and biofilm formation on medical devices and biomaterials is the quantitative evaluation of the actual bacterial number. As a matter of fact, bacterial count has deep implications in diagnostic and therapeutic treatments, as well as in quality controls [20–26]. The standard method used to evaluate the number of bacteria based on determination of colony forming units (CFUs) can be considered fully appropriate only when bacteria are in planktonic lifestyle but it is unreliable to count bacteria adherent and in biofilm lifestyle [27]. Therefore, the number of adherent bacteria as well as that of bacteria in biofilm lifestyle on SWCNTs-coated surfaces was evaluated using BioTimer Assay (BTA) which allows easily counting bacteria in adherent and biofilm lifestyle without sample manipulation [20, 21, 28, 29]. 

## 2. Materials and Methods

### 2.1. Bacterial Strains and Culture Media

*Streptococcus mutans* ATCC 25175^T^ and *Pseudomonas aeruginosa* ATCC 15692 (PAO1) were maintained in Trypticase soy broth (TSA; Difco Laboratories, MD, USA) with glycerol (25%) at −80°C and checked for purity on Columbia CNA agar (Difco) with 5% red sheep cells and TSA, respectively, before use. *S. mutans* and *P. aeruginosa* were grown in 1% sucrose-brain hearth infusion (BHI; Oxoid Ltd., UK) and BHI (Oxoid) broth, respectively, at 37°C without agitation for 18 to 24 hours. 

### 2.2. Single Wall Carbon Nano Tubes-Coated Glass Surfaces

Commercial glass beads (GBs) with 5 mm of diameter and commercial coverslips (CSs) were used as surfaces for the experiments, either as uncoated or coated with single wall carbon nanotubes (SWCNTs) films. 

SWCNTs coated GBs and CSs (namely, SWCNTs-GBs and SWCNTs-CSs, resp.) were produced by first cleaning the purchased GBs and CSs for 30 min in a solution composed of one-third of H_2_O_2_ (30%) and two-thirds H_2_SO_4_ (18 M). GBs and CSs were subsequently washed with distilled water and dried under a N_2_ flow. Immediately after cleaning, GBs and CSs were coated with SWCNTs. To this aim, commercial SWCNTs (Cheap Tubes Inc., according to the manufacturer, nanotubes were produced by catalytic CVD technique, with purity >90% and outer diameter 1-2 nm) were previously dispersed in a CHCl_3_ solution and then deposited on the two different types of substrates by drop casting. 

The overall quality of the realized surfaces was verified before and after the sterilization process by AFM imaging, as discussed below. 

### 2.3. Sterilization of Uncoated and SWCNTs-Coated Glass Surfaces

Both uncoated and SWCNTs-coated glass surfaces were sterilized by autoclaving at 121°C for 15 min and immersing them in 3% H_2_O_2_ solution for 10 min. After H_2_O_2_ sterilization, uncoated and SWCNTs-coated glass surfaces were washed three times in sterile distilled water to remove residual H_2_O_2_. 

### 2.4. Bacterial Adhesion and Biofilm Formation

To obtain bacterial adhesion and biofilm development, overnight sucrose-BHI and BHI cultures of *S. mutans *and* P. aeruginosa,* respectively, were incubated for 3 and 24 hours at 37°C in the presence of uncoated and SWCNTs-coated glass surfaces. 

### 2.5. Detection of Bacterial Colonization on Uncoated and SWCNTs-Coated Glass Surfaces

After incubation, uncoated and SWCNTs-coated glass surfaces were washed three times in sterile saline (0.9% NaCl) solution and the bacterial number was estimated by Biotimer Assay (BTA) [20, 21, 28, 29]. BTA employs different specific reagents for *Streptococcus *and* Pseudomonas* genera. BioTimer-phenol red reagent (BT-PR), previously set up to count fermenting *Staphylococcus* spp. biofilm [20] was used to count *S. mutans, *a fermenting bacterium. The final BT-PR appeared clear and red (pH 7.2) [20]. BioTimer-resazurin reagent (BT-RZ) developed to count *P. aeruginosa*, a nonfermenting bacterium [29], was prepared as follows. 3.7 g BHI was dissolved in 940 mL of distilled water. After sterilization for 15 minutes at 121°C, the reagent was supplemented with 50 mL of 10% sterile glucose solution and 10 mL of 0.1% sterile resazurin solution (Sigma-Aldrich, Italy). The final BT-RZ reagent appeared clear and blue (pH 7.0) [29]. 

**Figure 1:**
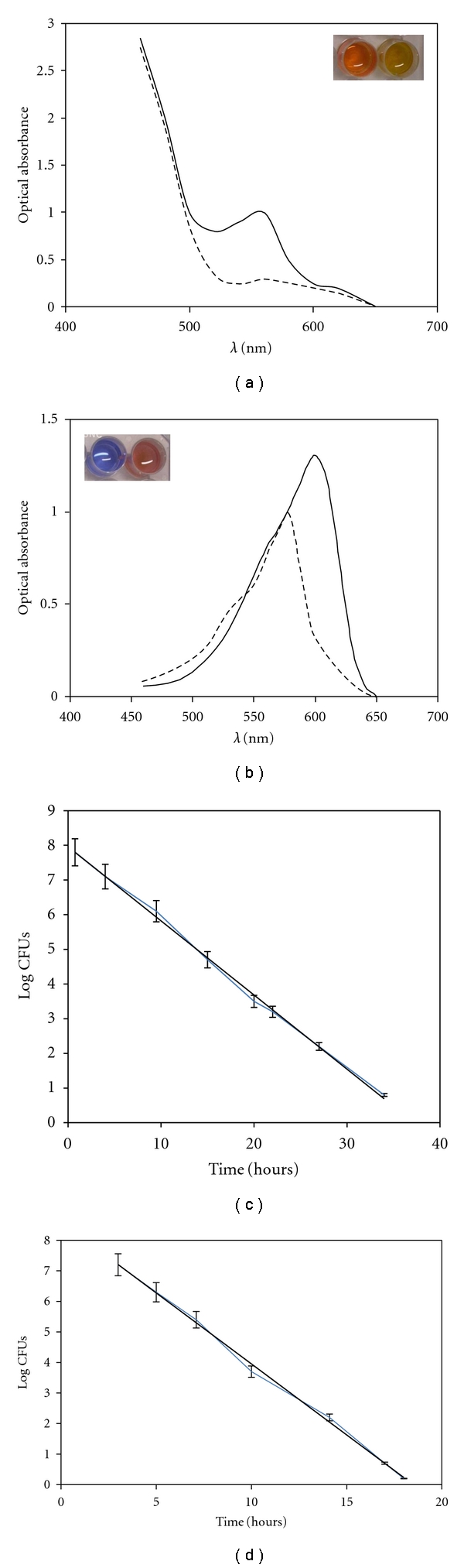
Color switch of BTA reagents and BTA correlation lines, (a)-(b) optical absorbance expressed as optical density versus the wave length in the visible region for BT-PR (a) and BT-RZ (b) reagents. Solid line: optical absorbance of BTA reagent before the switch; dotted line: optical absorbance of BTA reagent after the switch. Inserts: (a) BT-PR reagent color before (left) and after (right) the switch (red and yellow, resp.); (b) BT-RZ reagent color before (left) and after (right) the switch (blue and pink, resp.). (c)-(d) correlation lines correlate the time (hours) for color switch of BT-PR (c) and BT-RZ (d) reagents and log of the initial number N^0^ of planktonic *Streptococcus mutans * ATCC 25175^T^ (c) and *Pseudomonas aeruginosa *ATCC 15692 (d). Correlation lines were described by the following linear equations: *y* = −0.21 32*x* + 7.9597 and *r* ^2^ = 0.9899 for *S. mutans* (c) and *y* = −0.4675*x* + 8.5421 and *r* ^2^ = 0.9968 for *P. aeruginosa* (d).

BTA measures microbial metabolism: the time required for color switch of BTA reagents (i.e., BT-PR: red-to-yellow; BT-RZ: blue-to-pink; Figure 1), due to bacterial metabolism, is correlated to initial bacterial concentration. Therefore, the time required for color switch determines the number of bacteria present in a sample at time 0 through a correlation line. To draw the correlation lines to count *S. mutans* and *P. aeruginosa, *serial two-fold dilutions of overnight broth cultures in 1 mL of BT-PR and BT-RZ reagents, respectively, were performed in 24-well plates (BD, Italy) and simultaneously counted using CFU method. The time required for color switching of the inoculated BT-PR and BT-RZ reagents was recorded and plotted versus the corresponding CFU values (Figure 1). The equations and the linear correlation coefficients describing the correlation lines were calculated for each microorganism on the whole dataset and were *y* = −0.21 32*x* + 7.9597 and *r* ^2^ = 0.9899 for *S. mutans* and *y* = −0.4675*x* + 8.5421 and *r* ^2^ = 0.9968 for *P. aeruginosa*. Colonized GBs were immersed in 1 mL of the specific BTA reagent. The time required for color switching of the inoculated BT-PR and BT-RZ reagents was recorded and used to evaluate the number of *S. mutans* and *P. aeruginosa, *respectively, through the specific correlation line. As the correlation lines correlated the time for color switch of BTA reagents with the number of planktonic CFUs, the number of adherent and biofilm *S. mutans* and* P. aeruginosa* was expressed as planktonic-equivalent CFUs (PE-CFUs) [19, 20].

To evaluate the biomass of adherent and biofilm bacteria on uncoated and SWCNTs-coated glass surfaces, crystal violet detection protocol was used [11].

To visualize bacterial colonization on uncoated and SWCNTs-coated glass surfaces, atomic force microscopy (AFM) and BacLight LIVE/DEAD epifluorescent microscopy were employed [11, 18, 19]. AFM morphological characterization has been performed using a standard apparatus (Solver P47H, NT-MDT, Russian Federation) equipped with standard Silicon cantilevers. Images were collected in standard AFM semicontact mode in air and at room temperature. Concerning epifluorescence microscopy, sterile uncoated- or SWCNTs CSs were deposited on the bottom of 24-well flat microtiter plates (BD Falcon, Milan, Italy). *S. mutans *and* P. aeruginosa* were incubated at 37°C. After incubation, the coverslips were washed three times with distilled water and stained using the BacLight LIVE/DEAD viability probe (Molecular Probes) following manufacturer's instructions. After 15 min of incubation in the dark, viable (stained green) and nonviable cells (stained red) were observed by using epifluorescent optical microscopy (Leitz, Dialux 20 EB).

### 2.6. Statistics

All experiments were repeated at least five times to obtain mean values and standard deviations. Correlation lines were obtained by linear regression analysis, and linear correlation coefficients were calculated from the following equation:

(1) $$r=\frac{n\sum xy-\sum x\sum y}{\text{sqrt}\left(\left(n\sum x^{2}-{\left(\sum x\right)}^{2}\right)\left(n\sum y^{2}-{\left(\sum y\right)}^{2}\right)\right)}.$$

## 3. Results

### 3.1. Sterilization of Uncoated and SWCNTs CSs

To evaluate the efficiency of sterilization processes, the uncoated and SWCNTs CSs were treated by autoclaving and H_2_O_2_ solution (see Section 2 for details) and then immersed in BT-PR and BT-RZ reagents to control the absence of both fermenting and nonfermenting viable bacteria, respectively. BTA reagents not switched in the presence of sterilized uncoated and SWCNTS-CSs also after prolonged incubation (48 hours) demonstrating the absence of live bacterial cells (data not shown). 

**Figure 2:**
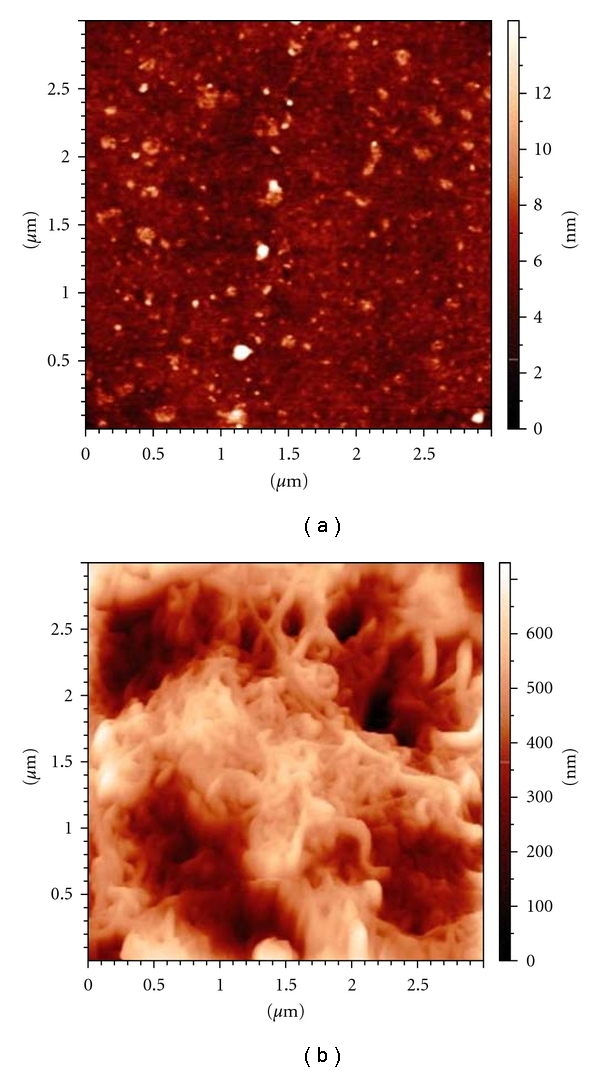
AFM images of (a) uncoated-CS and (b) SWCNTs CS substrates.

To evaluate the effect of the sterilization processes on nanocoated surfaces, SWCNTs CSs were observed by AFM before and after the sterilization processes and multiple random images were taken at various points of the samples surfaces. The microscopy images showed that the nanotubes formed a close and almost uniform film, thus demonstrating that the adopted nanocoating methodology was able to ensure a uniform nanocoated surface constituted by randomly entangled SWCNTs bundles. No significant differences were observed in the SWCNTs coating films before and after the sterilization process. In Figure 2, representative images of uncoated (Figure 2(a)) and SWCNTs CSs (Figure 2(b)) after sterilization procedures are shown. 

As the two sterilization processes gave comparable results, sterilization by autoclaving was employed in further experiments. 

### 3.2. S. mutans and P. aeruginosa Colonization of Uncoated and SWCNTs-GBs

To evaluate the adhesion ability of *S. mutans* and *P. aeruginosa*, different bacterial inoculum concentrations were used to colonize both uncoated and SWCNTs-GBs. After three hours of incubation, the number of adherent bacteria was estimated by BTA method (Table 1). Concerning *S. mutans*, the number of adherent bacteria depended on bacterial inoculum concentrations at least for inoculums ranging from 10^5^ to 10^7^ CFUs/mL (Table 1). Comparable values of adherent bacteria on uncoated and SWCNTs-GBs were recorded. Regarding *P. aeruginosa*, the number of adherent bacteria was partially related to inoculum concentrations. Like what was observed for *S. mutans*, the number of *P. aeruginosa* cells adhering on uncoated and SWCNTs-GBs was comparable. Concerning the bacterial biomass formed after three hours of incubation, similar values of eluted CV in all experimental conditions were recorded, thus indicating that biofilm was not developed during the incubation (Table 1). 

In order to allow the development of microbial biofilm, uncoated and SWCNTs-coated GBs were incubated in the presence of *S. mutans* and *P. aeruginosa* for 24 hours. Unlikely to what observed after three hours of incubation, the number of adherent bacteria was not influenced by the inoculum concentrations and it was the same on both uncoated and SWCNTs-GBs. In particular, the number of *S. mutans* and *P. aeruginosa* in biofilm was 3.4 ± 0.5 × 10^8^ and 4.3 ± 0.4 × 10^7^, respectively. To control the biofilm development on both uncoated and SWCNTs-GBs, colonized GBs were stained with CV. The results demonstrated that biofilm was developed at the same extent both on uncoated and SWCNTs-GBs. As a matter of fact, the values of CV eluted from uncoated and SWCNTs-GBs colonized with *S. mutans* were 0.7851 ± 0.1856 and 0.8331 ± 0.235, respectively, and those from uncoated and SWCNTs-GBs colonized with* P. aeruginosa *were 0.7515 ± 0.216 and 0.8131 ± 0.314, respectively. In control experiments, sterile uncoated and SWCNTs-GBs were stained with CV. The value of eluted crystal violet from both uncoated- and SWCNTs-GBs was 0.005 ± 0.001.

### 3.3. Microscopy

**Figure 3:**
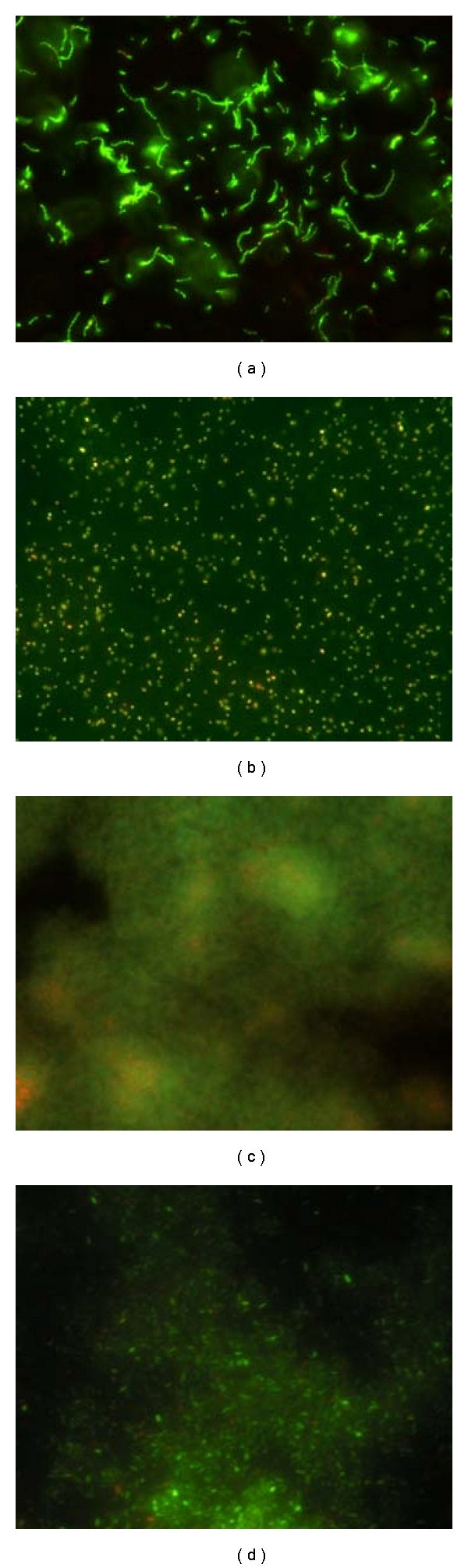
Epifluorescent optical microscopy of SWCNTs CSs colonized by *Streptococcus mutans. *ATCC 25175^T^ and *Pseudomonas aeruginosa *ATCC 15692* S. mutans * (a) and *P. aeruginosa *(b) adherent bacteria on SWCNTs CSs after 3 hours of incubation; *S. mutans * (c) and *P. aeruginosa *(d) biofilm on SWCNTs CSs after 24 hours of incubation.

To visualize adherent bacteria, uncoated and SWCNTs CSs were inoculated with 10^8^ UFC/mL and 10^9^ UFC/mL of *S. mutans* and *P. aeruginosa*, respectively. After 3 and 24 hours of incubation, the CSs were stained with LIVE/DEAD stain and observed using epifluorescent microscopy. Microscopy images showed that after 3 hours of incubation almost all adherent bacteria were alive both on uncoated and SWCNTs CSs. Representative images of adherent *S. mutans* and *P. aeruginosa* are shown in Figures 3(a) and 3(b), respectively. After 24 hours of incubation, large aggregates of live bacterial cells were surrounded by what is assumed to be polysaccharide matrix indicating biofilm lifestyle both on uncoated and SWCNTs CSs. Representative images of *S. mutans* and *P. aeruginosa* biofilm are shown in Figures 3(c) and 3(d), respectively. 

**Figure 4:**
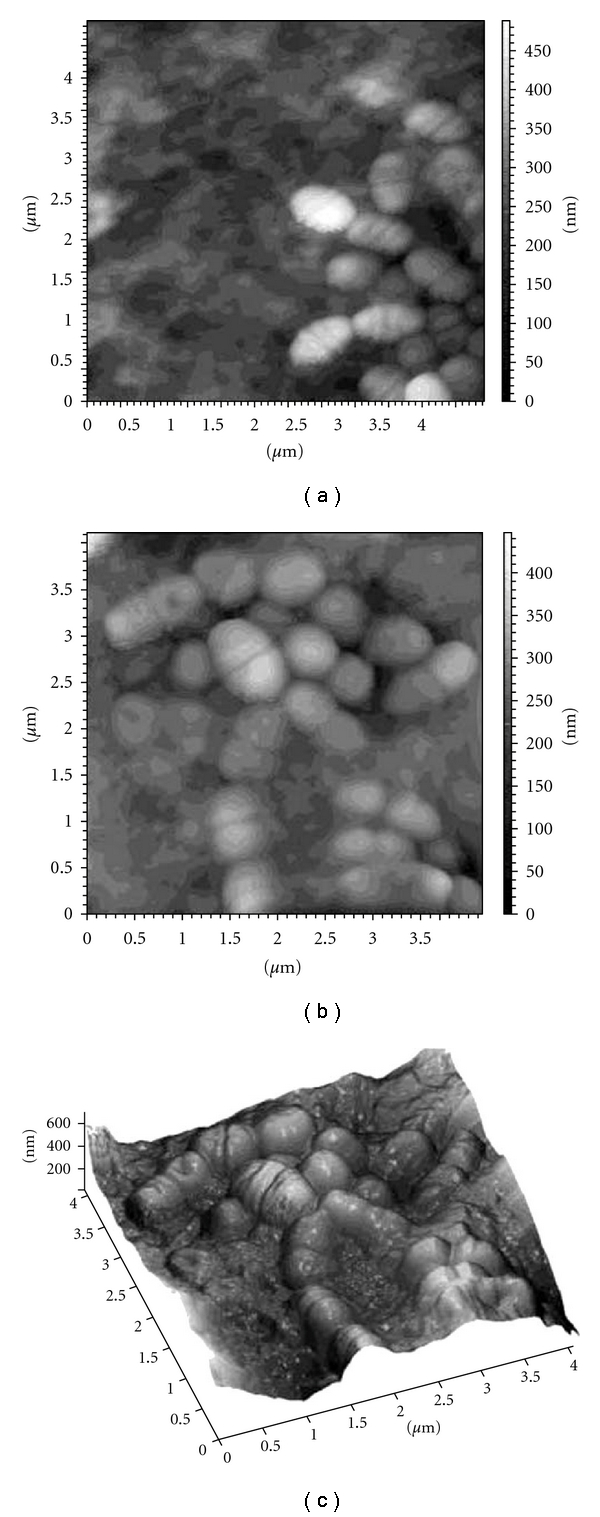
Atomic force microscopy images of *Streptococcus mutans* ATCC 25175^T^ colonizing single wall carbon nano tube-coated glass bead (SWCNT-GB). (a) SWCNT-GB colonization by *Streptococcus mutans* ATCC 25175^T^ after three hours of incubation (adherent *S. mutans*); (b) SWCNT-GB colonization by *S. mutans* ATCC 25175^ T^ after 24 hours of incubation (*S. mutans *biofilm); (c) three-dimensional view of the same area of (b).

**Figure 5:**
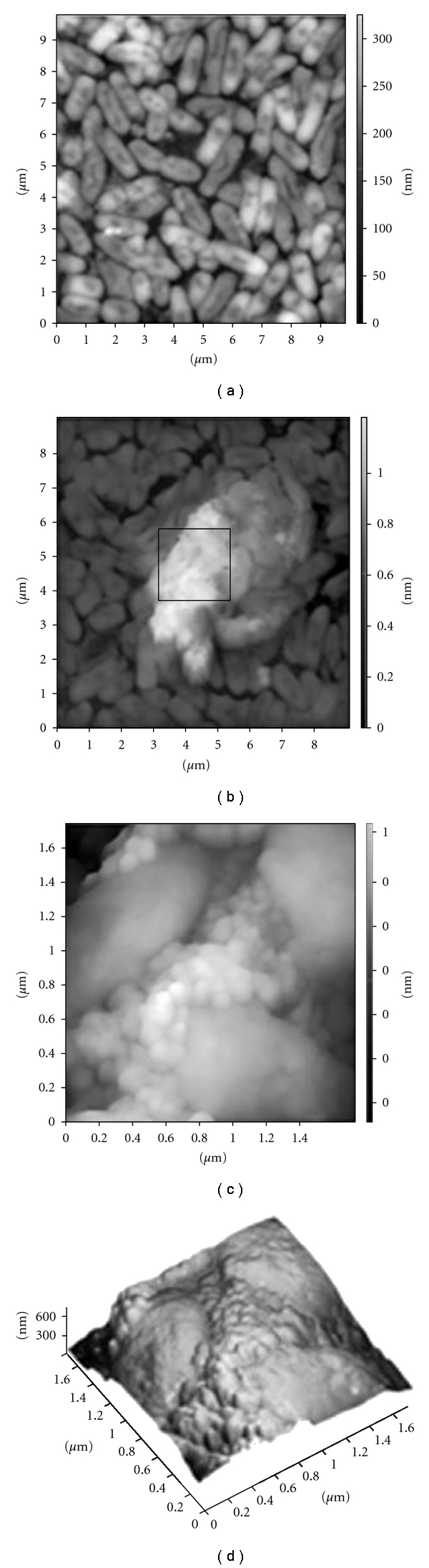
Atomic force microscopy images of *Pseudomonas aeruginosa* ATCC 15692 colonizing single wall carbon nanotube-coated glass bead (SWCNT-GB). (a) SWCNT-GB colonization by *Pseudomonas aeruginosa* ATCC 15692 after three hours of incubation (adherent bacteria); (b): SWCNT-GB colonization by *P. aeruginosa* ATCC 15692 after 24 hours of incubation (biofilm); (c) higher magnification of delimited area in (b) showing extracellular matrix between bacterial cells; (d) three-dimensional view of the same area of (c).

SWCNTs-GBs colonized for 3 and 24 hours were also analyzed by AFM. Despite the small area visualized, the multiple random AFM microscopy images are representative of microscopy observations carried out on SWCNTs-GBs colonized with *S. mutans* and *P. aeruginosa*, respectively (Figures 4 and 5). Both *S. mutans* and *P. aeruginosa* colonized the SWCNTs-GB surfaces after 3 hours of incubation (Figures 4(a) and 5(a)) and grew in biofilm lifestyle as suggested by the presence of the extracellular matrix in which bacteria were encased (Figures 4(b) and 4(c); Figures 5(b)–5(d)).

## 4. Discussion

The infections due to bacterial biofilm represent a pivotal problem in human health. Furthermore, biofilm infection becomes much more severe when biofilm is adherent and colonizes medical biomaterials [1–3]. Several biomaterials as those coated with antibacterial drugs have been developed and in vivo applied, but the bacterial colonization and biofilm development have been only partially inhibited [30]. The nanoscale sciences have led to the development of nano-structured materials able to counteract bacterial colonization and biofilm development [8, 9, 31]. The nanoscale structure of a material has an enormous impact on the properties of material itself [32]. As a matter of fact, the improved surface properties may play a key role in hindering medical devices- and implant-related infections. Therefore, the evaluation of adhesion ability as well as biofilm development of bacterial pathogens must be considered a crucial step in the design of new nano-structured biomaterials of medical interest. This implies the involvement of both physical and microbiological multidisciplinary skills. 

Here, we evaluated the ability of two bacterial pathogens to adhere and form biofilm on abiotic surfaces coated with SWCNTs. For this purpose, we have chosen two bacterial genera, involved in different medical devices infections: *S. mutans* representative of dental implant oral infections [12–14, 33] and *P. aeruginosa* of catheter-related infections [15–17, 34, 35]. 

Firstly, the efficiency and effect of sterilization processes were analyzed. We showed that the sterilization protocols (i.e., autoclaving or H_2_O_2_ treatment) were efficient and did not alter the overall quality of SWCNTs coating the glass surfaces. 

*S. mutans* and *P. aeruginosa* were able to adhere after three hours to uncoated and SWCNTs-coated surfaces showing comparable adhesion efficiency (Table 1). Moreover, similar live bacterial populations on uncoated and SWCNTs-coated surfaces were observed by epifluorescent microscopy (Figures 3, 4, and 5). When incubation was prolonged up to 24 hours, biofilm developed onto uncoated and SWCNTs-surfaces was similar (Figures 3, 4, and 5). The number of bacteria in biofilm onto uncoated GBs was similar to that formed on SWCNTs-GBs and similar live bacterial populations immersed in extracellular matrix were observed by epifluorescent and AF microscopy (Figures 3, 4, and 5). 

Taken on the whole, our results demonstrate that SWCNTs film coating glass surfaces did not affect both the adhesion and the biofilm formation ability of bacterial pathogens as *S. mutans *and *P. aeruginosa. *The inability of SWCNTs to inhibit biofilm formation has been previously reported by Deng et al. [36] who showed that both *Escherichia coli *and *Staphylococcus aureus* developed in biofilm lifestyle on CNTs aggregates. On the contrary, other authors show that CNTs inhibit bacterial adhesion, biofilm formation and exhibit bactericidal activity [11, 37]. These conflicting results may be due to the different experimental approaches. As a matter of fact, we employed two bacterial strains that are usually considered as a model for studying biofilm development and are well-known pathogens for humans. Moreover, it should be underlined that our experiments were carried out using SWCNTs-coated surfaces instead of SWCNTs dispersed in aqueous solution or in polymers [8, 36, 37].

## 5. Conclusion

Taking into account the multidisciplinary approach of nanotechnology and the fact that recently nanotechnology is developing very rapidly, a great care must be done concerning the new products to be employed for the human health. 

To study the putative antibacterial property of SWCNTs nano-structured surfaces, the quantitative evaluation of live adherent bacteria is mandatory. In this respect, our results demonstrate that SWCNTs-coated surfaces are not suitable to counteract *S. mutans *and* P. aeruginosa* adhesion and biofilm development.

## Figures and Tables

**Table 1:** Adhesion of *Streptococcus mutans* ATCC 25175^T^ and *Pseudomonas aeruginosa* ATCC 15692 on uncoated- and SWCNT-coated-GBs.

Bacteria	Inoculum (CFUs/mL)	Uncoated GBs^a^		SWCNT-GBs	
		Crystal violet (OD_570_)	Adherent bacteria (PE-CFUs)^b^	Crystal violet (OD_570_)	Adherent bacteria (PE-CFUs)
*S. mutans *	4.2 ± 0.8 × 10^8^	0.009 ± 0.001	3.0 ± 0.3 × 10^7^	0.007 ± 0.001	2.5 ± 0.4 × 10^7^
	4.2 ± 0.5 × 10^7^	0.006 ± 0.002	1.5 ± 0.5 × 10^7^	0.011 ± 0.001	2.0 ± 0.3 × 10^7^
	4.0 ± 0.6 × 10^6^	0.008 ± 0.001	2.0 ± 0.4 × 10^6^	0.006 ± 0.002	1.8 ± 0.3 × 10^6^
	3.2 ± 0.3 × 10^5^	0.006 ± 0.002	1.2 ± 0.3 × 10^5^	0.005 ± 0.001	1.0 ± 0.5 × 10^5^
	0	0.005 ± 0.001	0^c^	0.005 ± 0.002	0^c^

*P. aeruginosa *	2.4 ± 0.3 × 10^9^	0.008 ± 0.002	8.0 ± 0.9 × 10^6^	0.009 ± 0.002	8.0 ± 0.6 × 10^6^
	2.1 ± 0.4 × 10^7^	0.006 ± 0.000	6.4 ± 0.7 × 10^5^	0.010 ± 0.001	6.4 ± 0.6 × 10^5^
	1.2 ± 0.7 × 10^6^	0.009 ± 0.001	1.2 ± 0.5 × 10^5^	0.007 ± 0.002	1.2 ± 0.2 × 10^5^
	2.7 ± 0.2 × 10^5^	0.007 ± 0.002	3.2 ± 0.3 × 10^4^	0.006 ± 0.001	4.0 ± 0.3 × 10^4^
	0	0.005 ± 0.001	0^c^	0.005 ± 0.001	0^c^

^a^uncoated-GB: uncoated glass beads; SWCNT coated-GBs: glass beads coated with single wall carbon nano tubes; ^b^adherent *S. mutans* and *P. aeruginosa* were counted using BTA method. The number of adherent bacteria is expressed as planktonic-equivalent CFUs (PE-CFUs; see Section 2 for details); ^c^incubation of BTA was prolonged to 48 hours.
